# Supplementary material for: Natural variation of DROT1 confers drought adaptation in upland rice
Source: Nat Commun. 2022 Jul 23;13:4265. doi: 10.1038/s41467-022-31844-w (PMC9308802; doi:10.1038/s41467-022-31844-w)
Supplement: Supplementary file 1 — Supplementary Information [file 41467_2022_31844_MOESM1_ESM.pdf]

Natural variation of *DROT1* confers drought adaptation in upland rice

Sun *et al*.

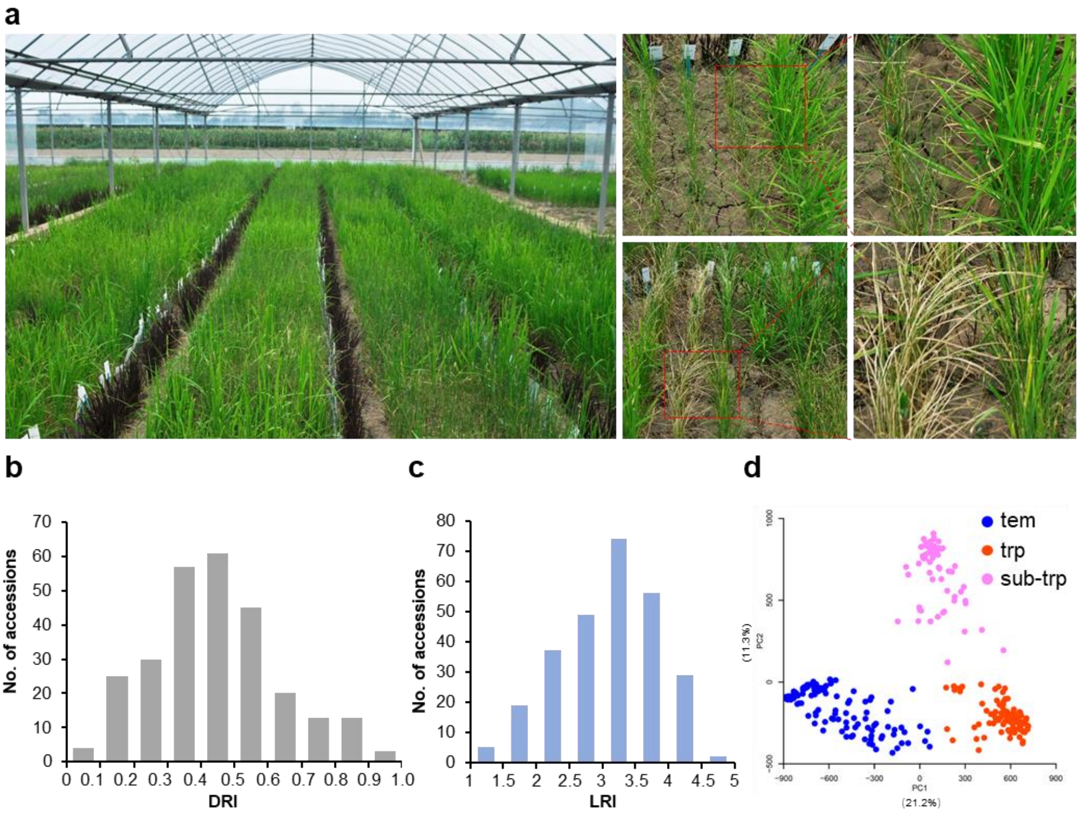

Supplementary Fig. 1. Phenotypic diversity and genetic structure of 271 *japonica* rice.

a Phenotypic investigation of 271 *japonica* rice accessions under drought stress. All materials were grown in soil under rain-proof shed (left). Under drought stress, the germplasms showed diversity in leaf curling (above center) and leaf color (lower center). The enlarged photos of red-framed area were attached to the right. **b, c** Phenotype distribution of DRI (**b**) and LRI (**c**) in 271 *japonica* rice varieties. **d** Principal component (PC) plot of different *japonica* subpopulations used for GWAS. Values in parentheses indicate percentage of variance explained by each principal component. Source data are provided as a Source Data file.

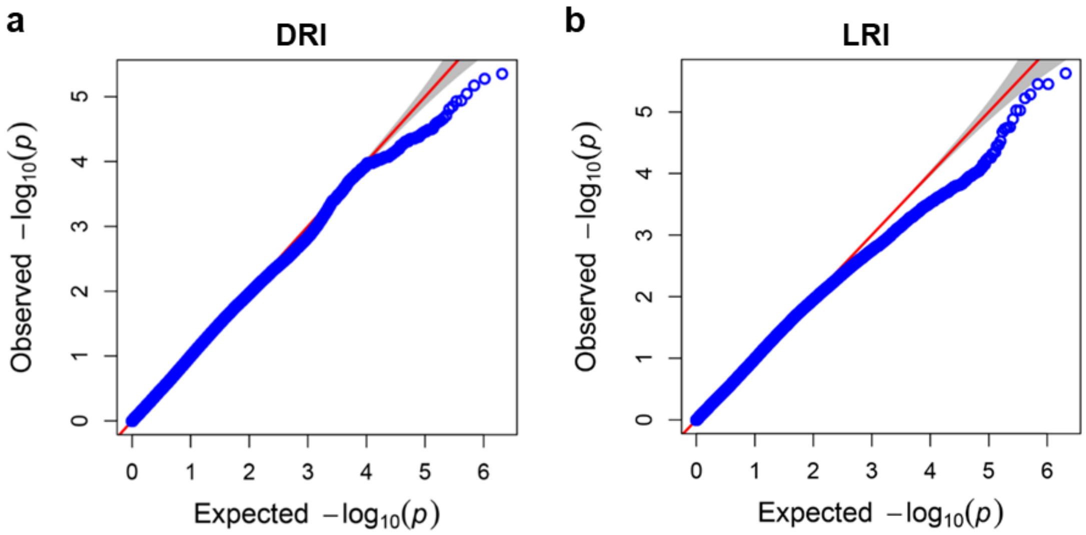

Supplementary Fig. 2. Quantile-quantile plots of observed versus expected –log$_{10}$*P* values of GWAS for DRI (a) and LRI (b).

The dotted lines show the 95% confidence interval for the QQ-plot under the null hypothesis of no association between the SNP and the trait. *P*-value was obtained from the *F*-test for testing H0: No association between the SNP and trait.

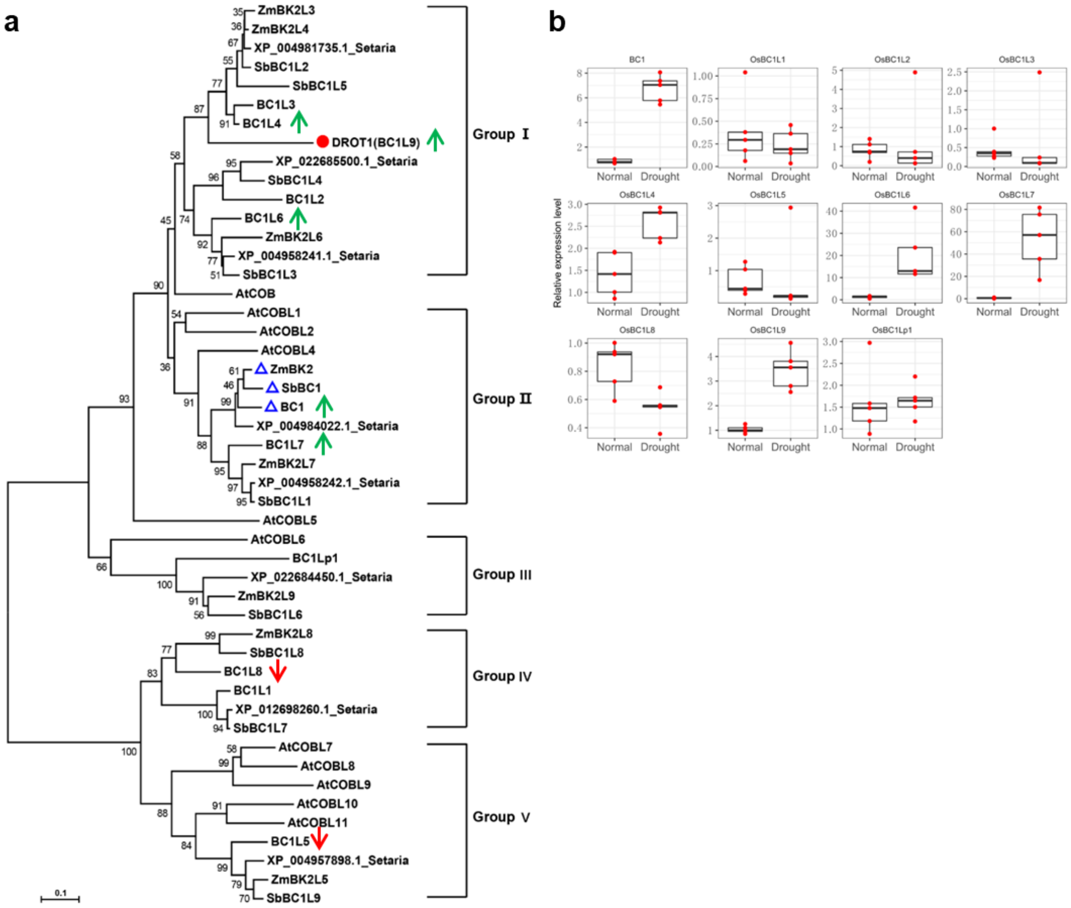

Supplementary Fig. 3. Conservation and diversification of DROT1 and its homologs in rice and other plants.

a Phylogenetic tree of COBRA-like proteins in monocot and dicot species. Neighbour-joining tree was constructed using MEGA 5.0. The numbers represent bootstrap (1,000 replicates). Red dot indicates the DROT1, and blue triangles represent three homologs in grass family that exhibit brittle stems and leaves in their loss-of-function mutants. Green arrows indicate induced expression of genes under drought stress, red arrows represent repressed expression of genes under drought stress according to **b**. **b** Expression patterns of rice COBRA family genes in Nipponbare under normal and drought stress conditions. Data are means ± s.d. (n = 5 biological replicates). In each box plot, the center line indicates the median, the edges of the box represent the first and third quartiles, and the whiskers extend to span a 1.5 interquartile range from the edges. Source data are provided as a Source Data file.

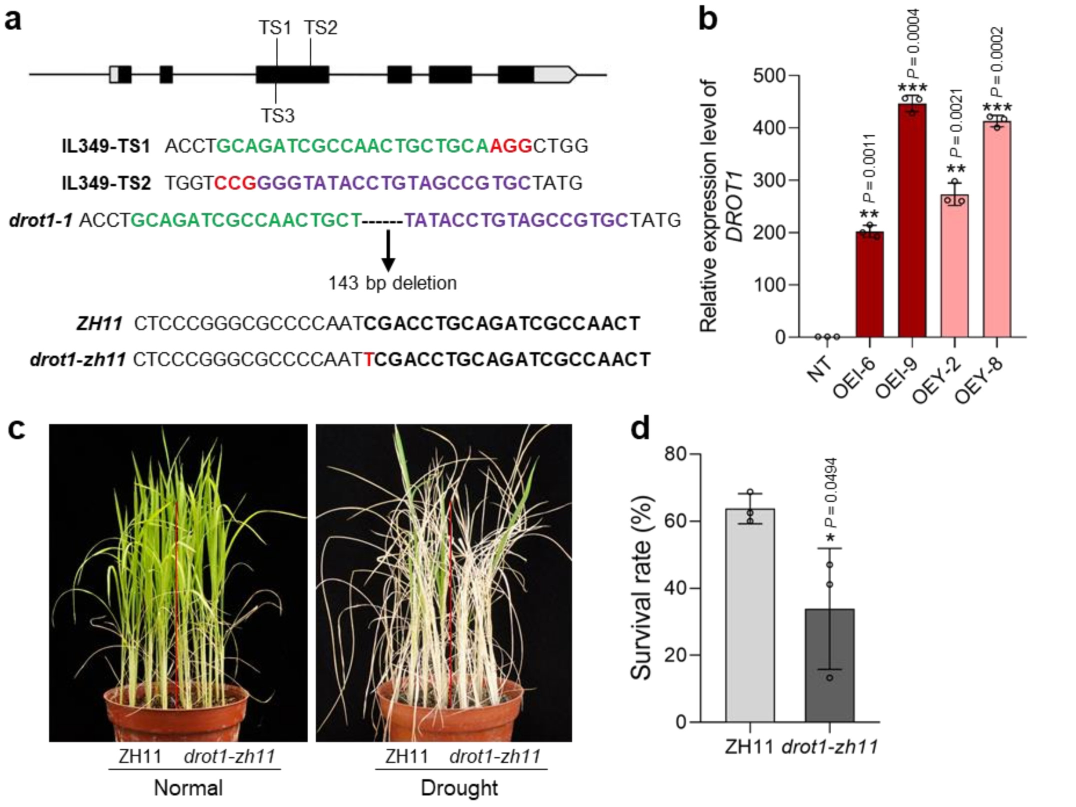

Supplementary Fig. 4. Identification of positive transgenic lines.

a Knockout of *DROT1* by CRISPR/Cas9 system. Three target sites were marked as TS1, TS2 and TS3 in the third exon. One mutant with a 143 bp deletion between TS1 and TS2 in IL349 background is shown as *drot1-1*. Another mutant with a 'T' insertion in TS3 of ZH11 background is named as *drot1-zh11*. **b** The expression of *DROT1* in overexpression lines. Data represent means ± s.d. (n=3 biological replicates). **c** Phenotypes of *drot1-zh11* and ZH11 under normal condition (left) or after drought stress (right). **d** Survival rate of ZH11 and *drot1-zh11* seedlings after re-watering. Data represent means ± s.d. (n=3 biological replicates). Asterisks indicate significant differences in two-tailed Student's *t*-tests (*P<0.05, **P<0.01, ***P<0.001). Source data are provided as a Source Data file.

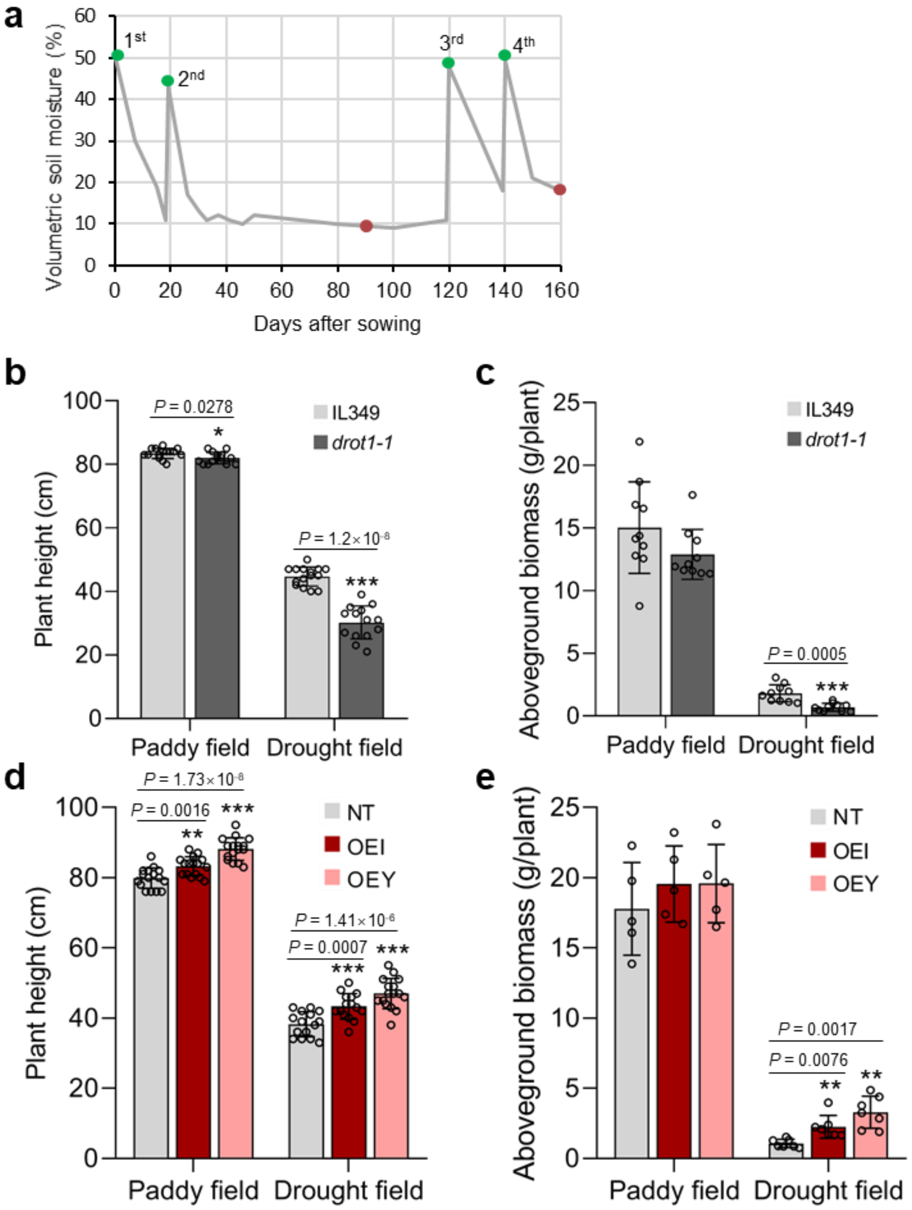

Supplementary Fig. 5. Plant height and aboveground biomass of control and transgenic lines grown for three months in paddy or severe drought field.

a Pattern of volumetric soil moisture in severe drought field during the life cycle. Green dots indicate the time points for water supply, red dots represent the time points for phenotypic investigation. **b** Plant height of *drot1-1* and IL349. Data represent means ± s.d. (n=15/14/15/14 plants, from left to right column). **c** Aboveground biomass of *drot1-1* and IL349. Data represent means ± s.d. (n=10 plants). **d** Plant height of NT, OEI and OEY. Data represent means ± s.d. (n=16/16/16/15/15/16 plants). **e** Aboveground biomass of NT, OEI and OEY. Data represent means ± s.d. (n=5/5/5/7/7/7 plants). Asterisks indicate statistical significance by two-tailed Student's *t*-tests (**P*<0.05, ***P*<0.01, ****P*<0.001). Source data are provided as a Source Data file.

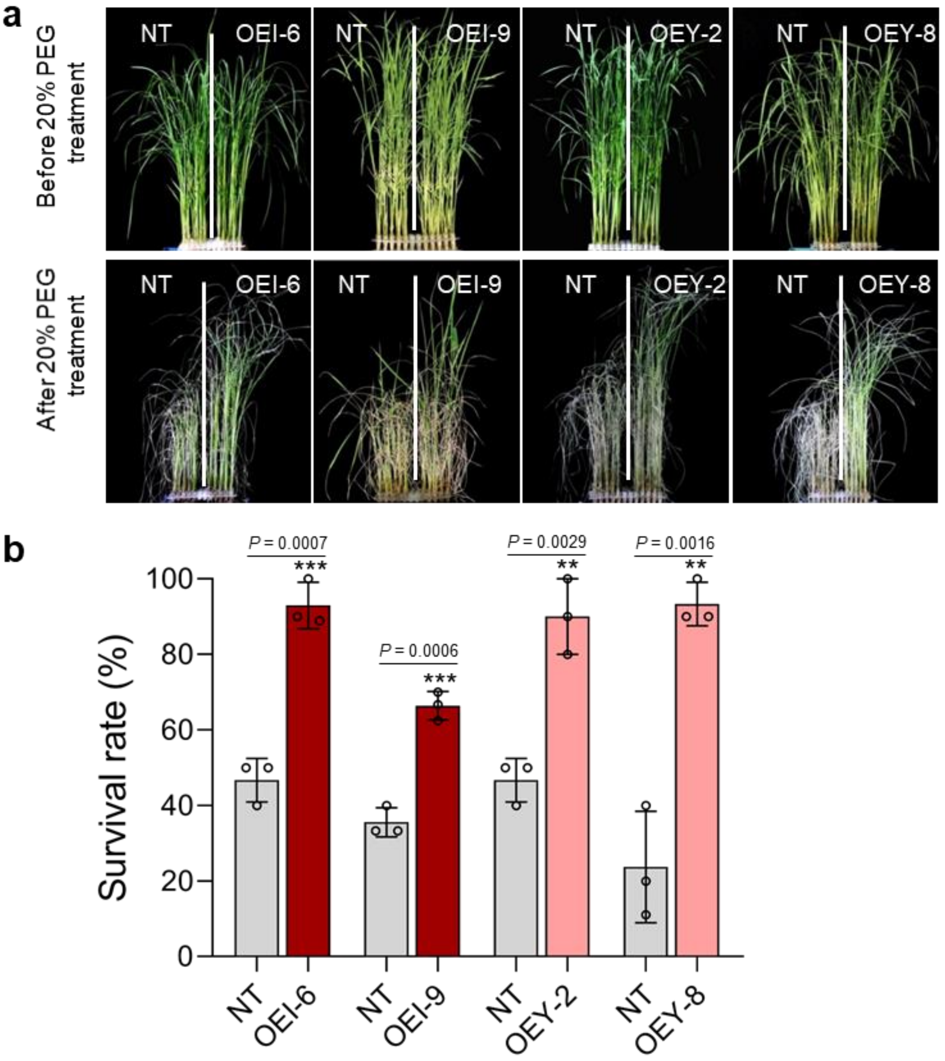

Supplementary Fig. 6. *DROT1* enhances stress tolerance under 20% PEG treatment.

a Phenotype of *DROT1* over expressing lines before and after 20% PEG treatment. Seedlings cultured in nutrient solution for one month were treated by 20% PEG6000 for three days and re-watered for seven days. **b** Statistical analysis of survival rate after re-watering. Data are means ± s.d. (n = 3 biological replicates). ***P*<0.001, ****P*<0.001, two-tailed Student's *t*-tests. Source data are provided as a Source Data file.

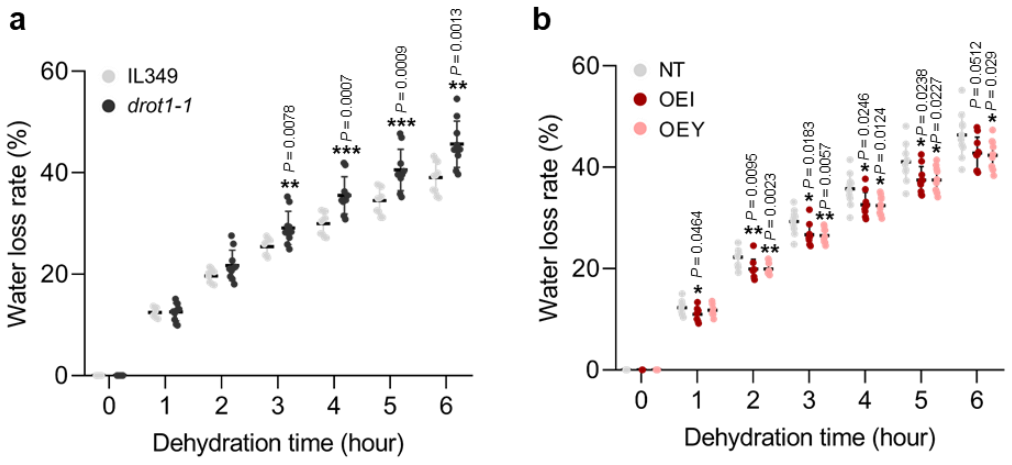

Supplementary Fig. 7. Water loss rate in flag leaves of control and transgenic plants.

a, b Rice leaves grown in the paddy field for 80 days were detached and placed on bench at room temperature (28°C). The leaves were weighed and values were recorded hourly in the next 6 consecutive hours. Data represent means ± s.d. (n=10 biological replicates). Asterisks indicate statistical significance by two-tailed Student's *t*-tests (**P*<0.05, ***P*<0.01, ****P*<0.001). Source data are provided as a Source Data file.

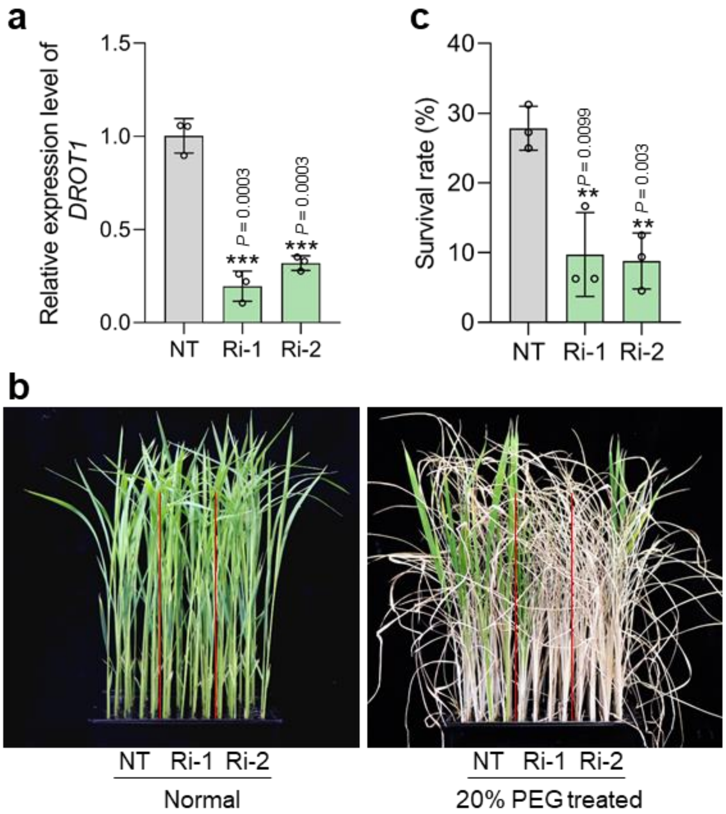

Supplementary Fig. 8. Down-regulation of *DROT1* reduces drought resistance.
a Expression of *DROT1* in NT and two independent RNAi lines. Data represent means ± s.d. (n=3 biological replicates). **b** Phenotype of NT and RNAi lines under normal growth conditions (left) and after 20% PEG6000 treatment for 5 days followed by re-watering for 10 days (right). **c** Statistical analysis of survival rate after re-watering. Data represent means ± s.d. (n=3 biological replicates). Asterisks indicate statistical significance by two-tailed Student's *t*-tests (***P*<0.01, ****P*<0.001). Source data are provided as a Source Data file.

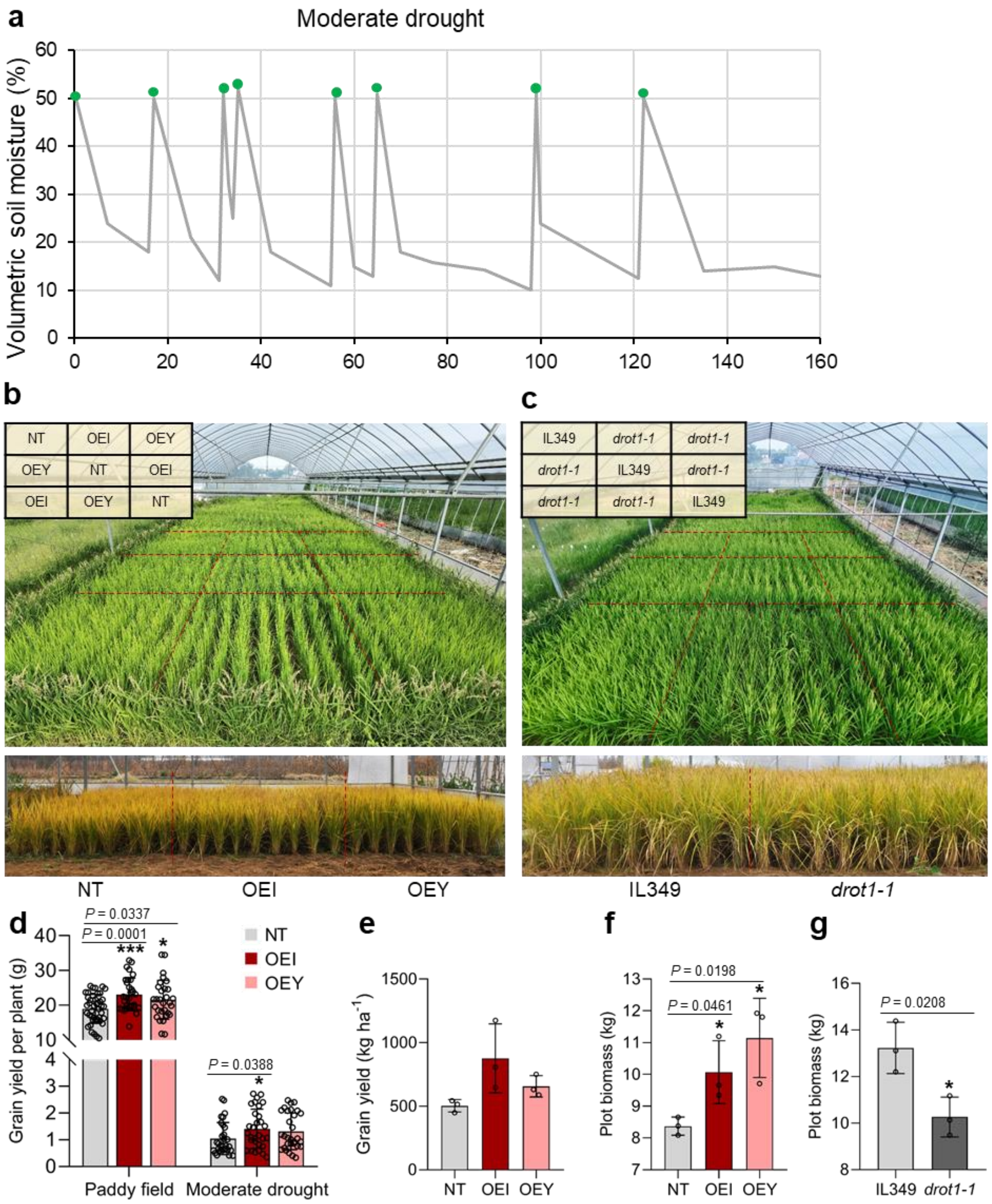

Supplementary Fig. 9. Evaluation of grain yield and yield-related traits under moderate drought conditions.

a Time course of volumetric soil moisture in moderate drought field throughout the life cycle. Green dots indicate the time points for water supply. **b, c** Side views of rice plants grown in rain-proof shed at flowering stage (upper) and mature stage (lower). **d** Grain yield per plant of NT and OE lines in paddy field and moderate drought field. Data represent means ± s.d. (n=43/32/30/30/30/30 plants). **e** Grain yield per hectare of NT and OE lines in moderate drought field. Values are means ± s.d. (n = 3 replications). **f, g** Comparison of plot biomass between NT and OE lines (**f**), IL349 and *drot1-1* **(g)** in moderate drought field. Data represent means ± s.d. (n=3 plots). Asterisks indicate statistical significance by two-tailed Student's *t*-tests (*P<0.05, ***P<0.001). Source data are provided as a Source Data file.

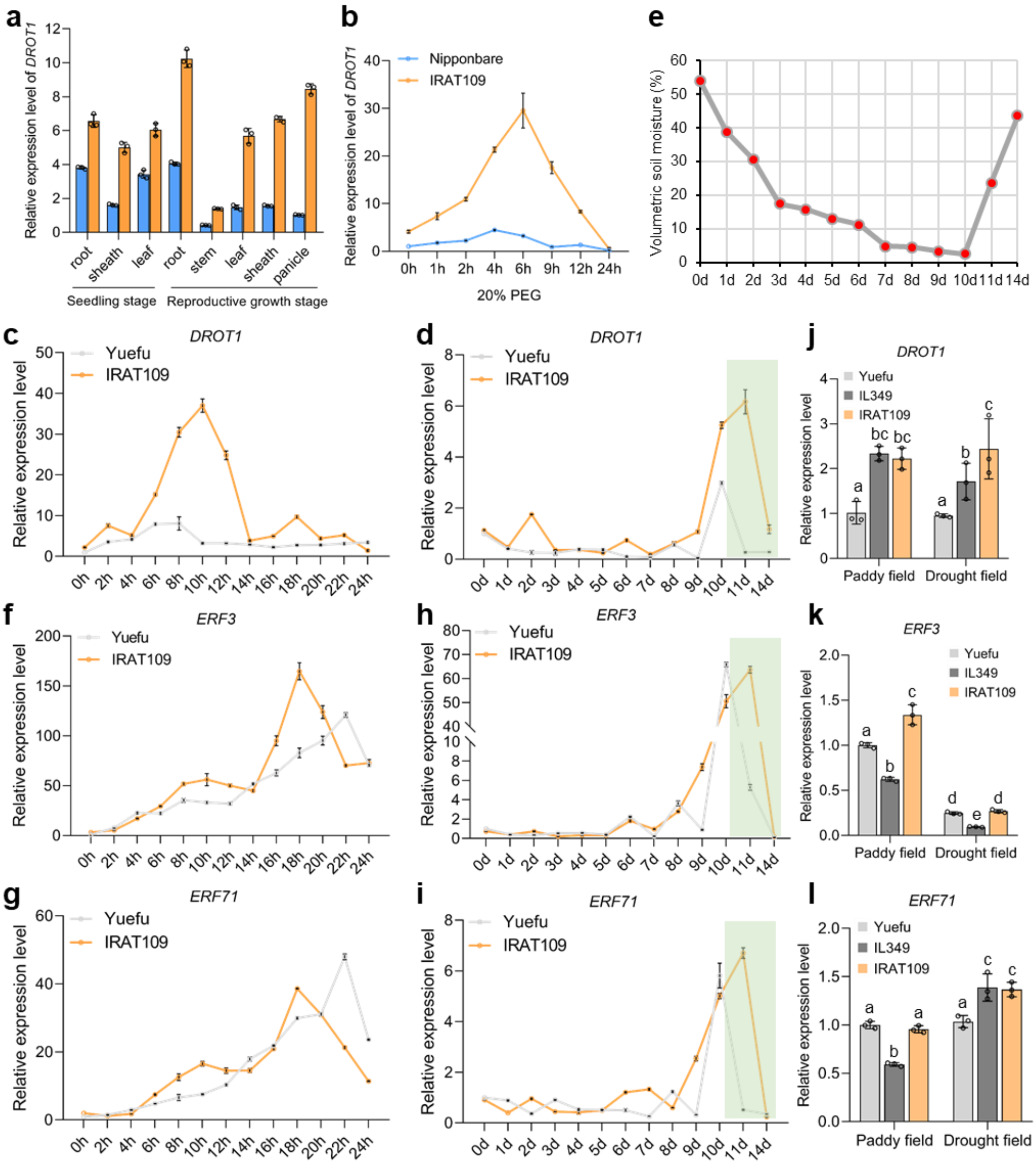

Supplementary Fig. 10. Expression patterns of *DROT1*, *ERF3*, *and ERF71*.

a Expression of *DROT1* in organs of IRAT109 and Nipponbare at seedling and reproductive stages. Data represent means ± s.d. (n=3 biological replicates). **b** Induced expression of *DROT1* under 20%PEG treatment. Error bars indicate the SD of three biological replicates. **c**, **d** Time-course expression of *DROT1* in Yuefu and IRAT109 under dehydrated stress (**c**) or drought stress without water supply in pots (**d**). Error bars indicate the SD of three biological replicates. **e** Time course of volumetric soil moisture in pots measured daily around 17:00 pm. **f-i** Time-course expression of *ERF3* and *ERF71* in Yuefu and IRAT109 under dehydrated stress (**f, g**) and/or drought stress without water supply in pots (**h**, **i**). Error bars indicate the SD of three biological replicates. **j-l** Expression of *DROT1* (**j**), *ERF3* (**k**) and *ERF71* (**l**) in Yuefu, IL349 and IRAT109 grown in paddy field and drought field. Leaf samples from accessions grown in paddy or drought field for 90 days were collected for RNA extraction and gene expression analysis. Data represent means ± s.d. (n=3 biological replicates). Different letters indicate statistically significant differences at P =0.05 by one-way ANOVA with Duncan test. Source data are provided as a Source Data file.

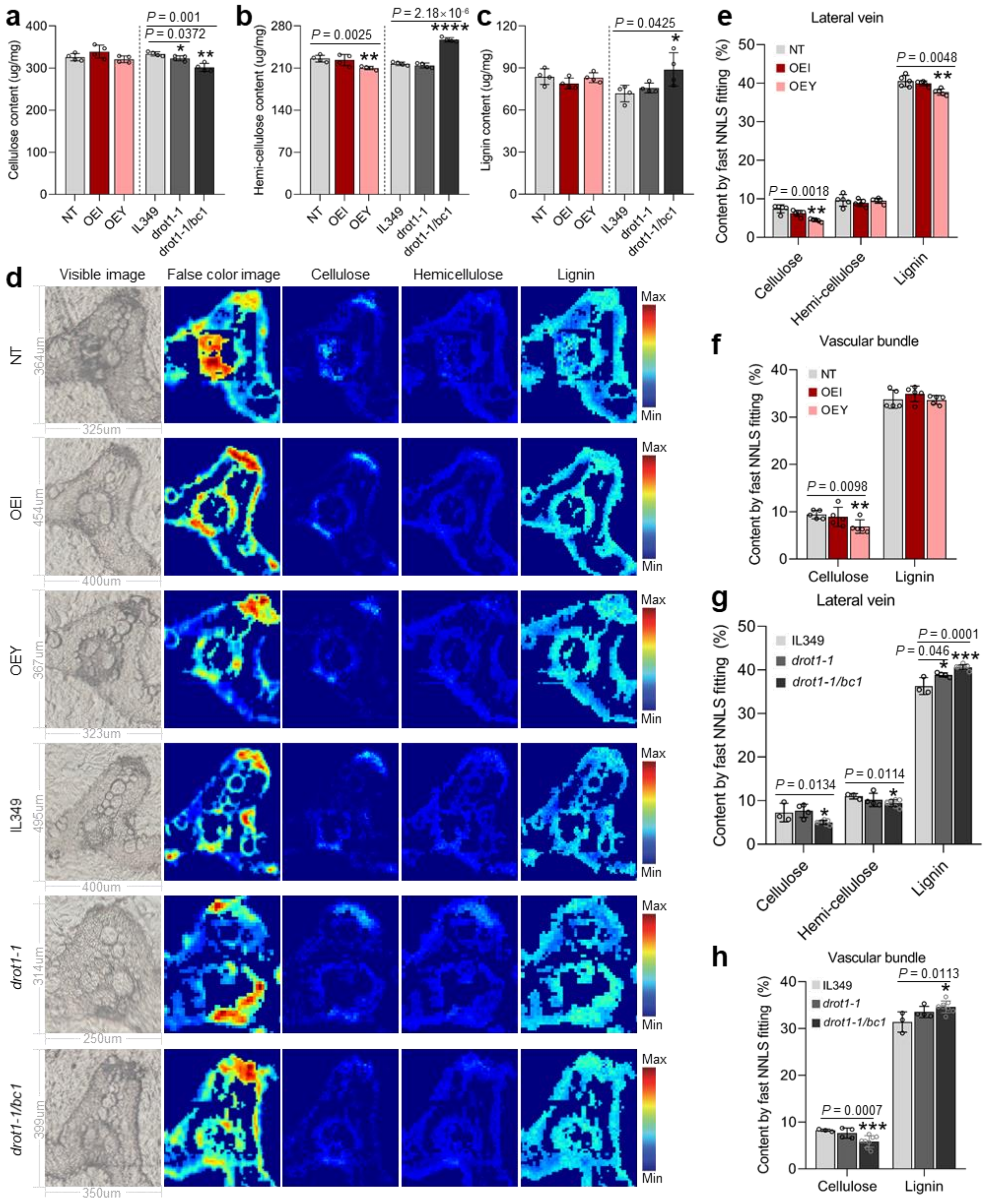

Supplementary Fig. 11. Cell wall components analysis in lateral veins of leaves grown under well-watered conditions by chemical and FTIR micro-spectroscopic method.

a-c The contents of cellulose **(a)**, hemi-cellulose **(b)** and lignin **(c)** in leaves determined by chemical method. Data are means ± s.d. (n=4 biological replicates). **d** Fast NNLS fitting images of cross sections in lateral veins of leaves grown in paddy field. **e-h** Semi-quantitative analysis of cell wall components. The contents of cellulose, hemi-cellulose and lignin were determined by fast NNLS fitting for lateral vein from **d** (**e, g**). The cellulose and lignin content were determined based on fast NNLS fitting for the segmented vascular bundle (**f**, **h**). The *drot1-1/bc1* double mutant having brittle leaves and stems was used to verify the accuracy of the FTIR analysis. It had a lower cellulose content and a higher lignin content in both the whole lateral vein and the vascular bundle compared with IL349, which is consistent with previous studies. Data are means ± s.d. (n= 3 biological replicates for **e** and **f**, n= 3/4/8/3/4/8/3/4/8 biological replicates for **g and h**). Asterisks indicate statistical significance by two-tailed Student's *t*-tests (**P*<0.05, ***P*<0.01, ****P*<0.001, *****P*<0.0001). Source data are provided as a Source Data file.

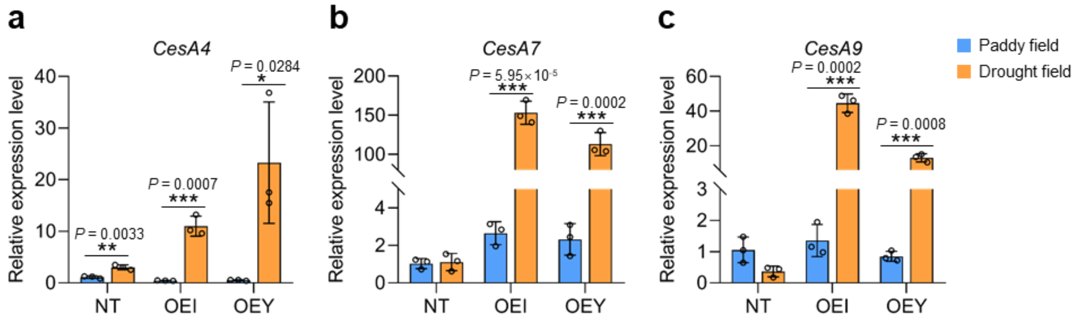

Supplementary Fig. 12. Induced expression of cellulose synthesis genes under drought field conditions.

Leaf tissues grown in paddy or drought field were sampled for RNA preparation. The expression of three cellulose synthesis genes, *CesA4* (**a**), *CesA7* (**b**), and *CesA9* (**c**), was investigated. Data are means ± s.d. (n=3 biological replicates). Asterisks indicate statistical significance by two-tailed Student's *t*-tests (**P*<0.05, ***P*<0.01, ****P*<0.001). Source data are provided as a Source Data file.

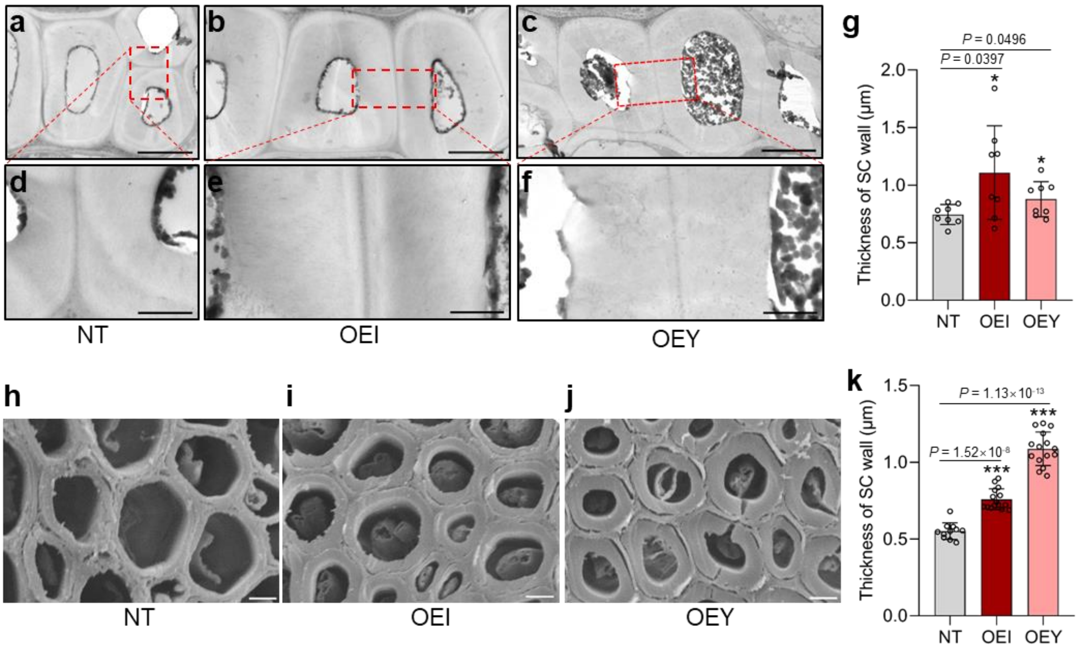

Supplementary Fig. 13. Thickened sclerenchyma cell walls in leaves and stems of OE lines.
a-c TEM images of sclerenchyma cells from 45-days-old leaves of the indicated rice plants. Bar=2 um. **d-f** Magnification of the area framed by red boxes in **a-c.** Bar=0.5 um. **g** Quantification of sclerenchyma cell wall thickness indicated in **a-c**. Data are means ± s.d. (n = 8 cells). **h-j** SEM images of sclerenchyma cells from the internodes of the indicated plants at flowering stage. Bar=2 um. **k** Quantification of sclerenchyma cell wall thickness indicated in **h-j.** Data are means ± s.d. (n = 15 cells). Asterisks indicate statistical significance by two-tailed Student's *t*-tests (**P*<0.05, ****P*<0.001). Source data are provided as a Source Data file.

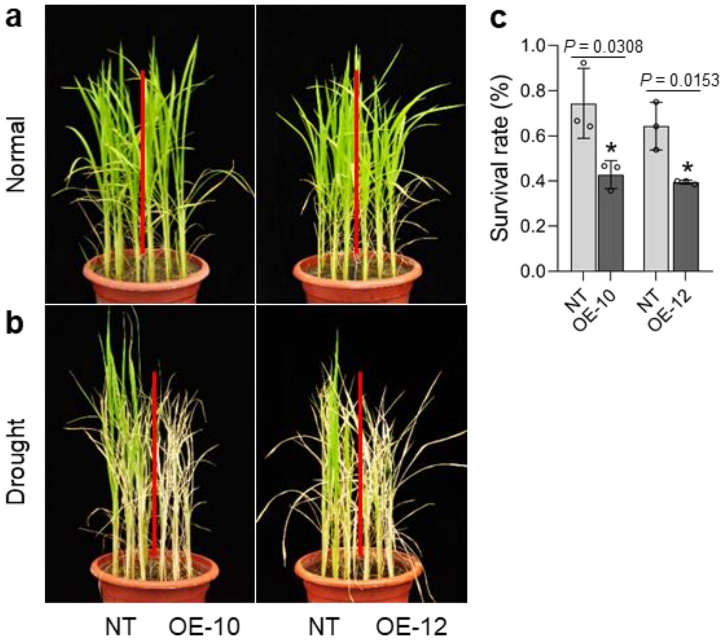

Supplementary Fig. 14. *ERF3* negatively regulates drought resistance in rice.

a, **b** Drought treatment of *ERF3*-overexpressing lines and NT. The seedlings grown for four weeks under normal conditions were photographed (**a**) and subsequently treated by drought stress for 15 days, followed by re-watering for 10 days (**b**). Two independent transgenic lines were used for this test. NT, non-transgenic lines. **c** Statistical analysis of survival rate after re-watering. Data are means ± s.d. (n = 3 biological replicates). Asterisks indicate statistical significance by two-tailed Student's *t*-tests, *P<0.05. Source data are provided as a Source Data file.

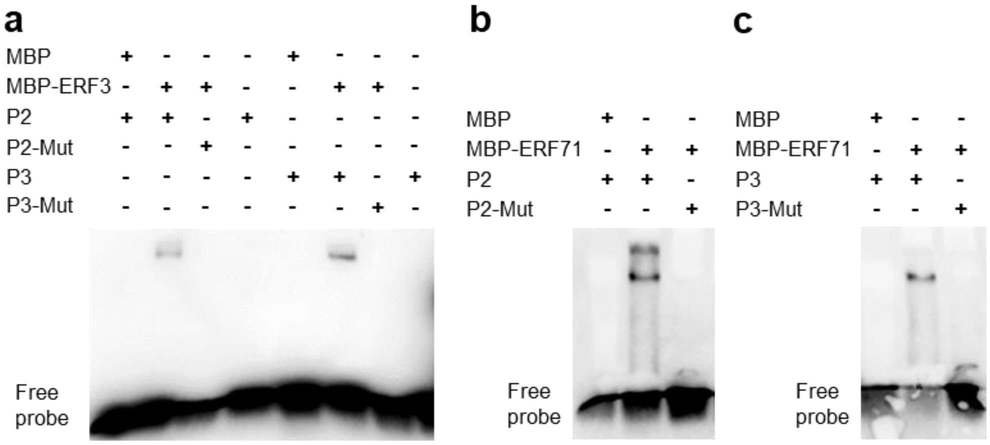

Supplementary Fig. 15. Examination of the ERFs binding motif at P2 and P3 in the promoter of *DROT1*.

a EMSA shows ERF3 can also bind the GCC box at P2 and P3. **b, c** ERF71 binds the GCC box at P2 **(b)** and P3 **(c).** n =4 independent experiments.

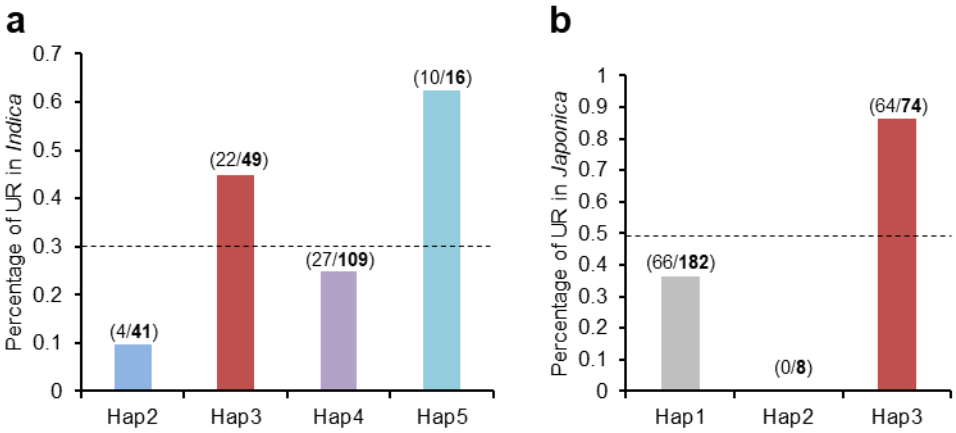

Supplementary Fig. 16. Proportion of upland rice in different haplotypes of *indica* and *japonica*.

a Percentage of upland rice in different haplotype of *indica.* Dashed line indicates the average proportion of upland rice (UR) in full *indica* population. **b** Percentage of upland rice in different haplotype of *japonica*. Dashed line indicates the average proportion of upland rice in full *japonica* population. The numbers in parentheses indicate the number of accessions (number of upland rice/ **total number of cultivar**).

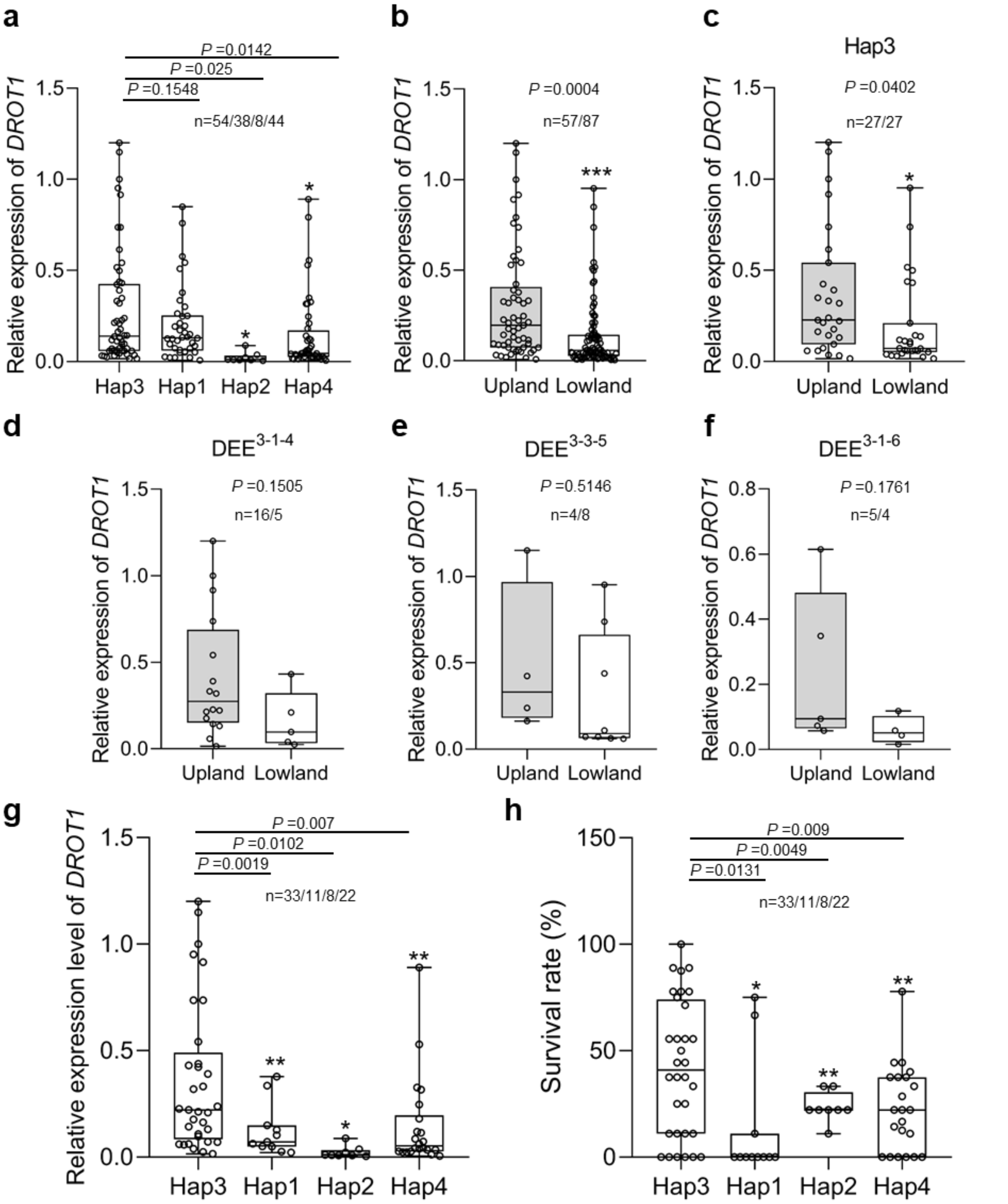

Supplementary Fig. 17. *DROT1* expression and survival rate of accessions with different haplotypes in rice germplasm.

a Expression of *DROT1* in accessions with different *DROT1* haplotypes regardless of haplotypes of ERF regulators. **b-f** Expression of *DROT1* in upland and lowland rice. **g, h** Expression of *DROT1* (**g**) and survival rate (**h**) in accessions with different haplotypes of *DROT1* which were classified based on the accessions used in Fig.7g and 7h. In each box plot, the center line indicates the median, the edges of the box represent the first and third quartiles, and the whiskers extend to span a 1.5 interquartile range from the edges. The significant differences were determined by two-tailed Student's *t*-tests and *P* values were indicated (*$P<0.05$, **$P<0.01$, ***$P<0.001$). Source data are provided as a Source Data file.

a

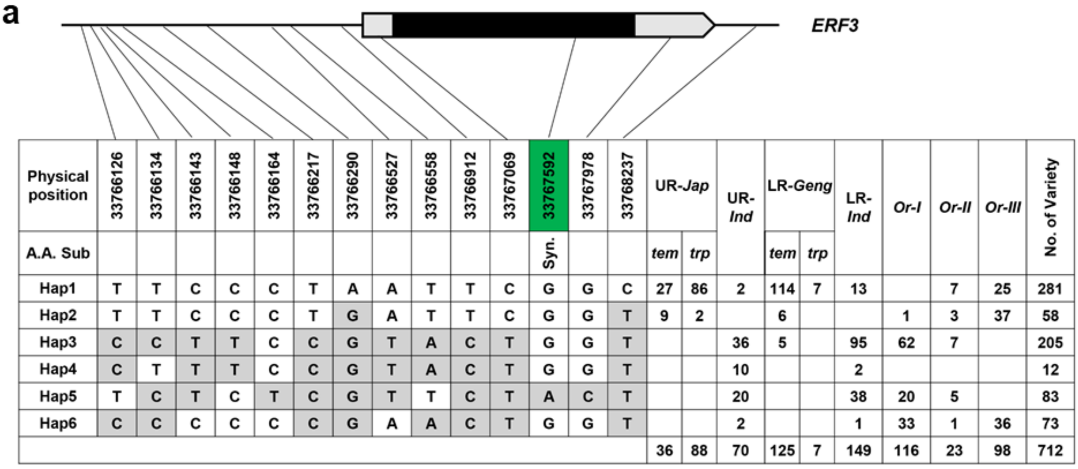

Physical position	33766126	33766134	33766143	33766148	33766164	33766217	33766290	33766527	33766558	33766912	33767069	33767592	33767978	33768237	UR-*Jap*		UR-*Ind*	LR-*Geng*		LR-*Ind*	*Or-I*	*Or-II*	*Or-III*	No. of Variety
A.A. Sub												Syn.			*tem*	*trp*		*tem*	*trp*					
Hap1	T	T	C	C	C	T	A	A	T	T	C	G	G	C	27	86	2	114	7	13		7	25	281
Hap2	T	T	C	C	C	T	G	A	T	T	C	G	G	T	9	2		6			1	3	37	58
Hap3	C	C	T	T	C	C	G	T	A	C	T	G	G	T			36	5		95	62	7		205
Hap4	C	T	T	T	C	C	G	T	A	C	T	G	G	T			10			2				12
Hap5	T	C	T	C	T	C	G	T	T	C	T	A	C	T			20			38	20	5		83
Hap6	C	C	C	C	C	C	G	A	A	C	T	G	G	T			2			1	33	1	36	73
															36	88	70	125	7	149	116	23	98	712

b

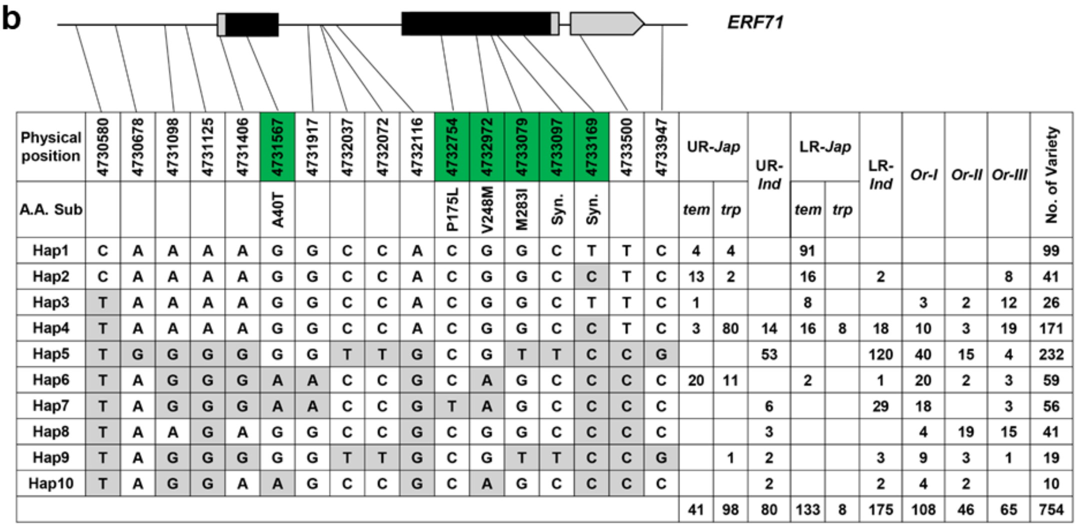

Physical position	4730580	4730678	4731098	4731125	4731406	4731567	4731917	4732037	4732072	4732116	4732754	4732972	4733079	4733097	4733169	4733500	4733947	UR-*Jap*		UR-*Ind*	LR-*Jap*		LR-*Ind*	*Or-I*	*Or-II*	*Or-III*	No. of Variety
A.A. Sub						A40T					P175L	V248M	M283I	Syn.	Syn.			*tem*	*trp*		*tem*	*trp*					
Hap1	C	A	A	A	A	G	G	C	C	A	C	G	G	C	T	T	C	4	4		91						99
Hap2	C	A	A	A	A	G	G	C	C	A	C	G	G	C	C	T	C	13	2		16		2			8	41
Hap3	T	A	A	A	A	G	G	C	C	A	C	G	G	C	T	T	C	1			8			3	2	12	26
Hap4	T	A	A	A	A	G	G	C	C	A	C	G	G	C	C	T	C	3	80	14	16	8	18	10	3	19	171
Hap5	T	G	G	G	G	G	G	T	T	G	C	G	T	T	C	C	G			53			120	40	15	4	232
Hap6	T	A	G	G	G	A	A	C	C	G	C	A	G	C	C	C	C	20	11		2		1	20	2	3	59
Hap7	T	A	G	G	G	A	A	C	C	G	T	A	G	C	C	C	C			6			29	18		3	56
Hap8	T	A	A	G	A	G	G	C	C	G	C	G	G	C	C	C	C			3				4	19	15	41
Hap9	T	A	G	G	G	G	G	T	T	G	C	G	T	T	C	C	G		1	2			3	9	3	1	19
Hap10	T	A	G	G	A	A	G	C	C	G	C	A	G	C	C	C	C			2			2	4	2		10
																		41	98	80	133	8	175	108	46	65	754

Supplementary Fig. 18. Haplotype analysis of *ERF3* and *ERF71*.

a Haplotype of *ERF3*. **b** Haplotype of *ERF71*. Nucleotide variations in the coding region are labeled in green boxes. Syn., synonymous.

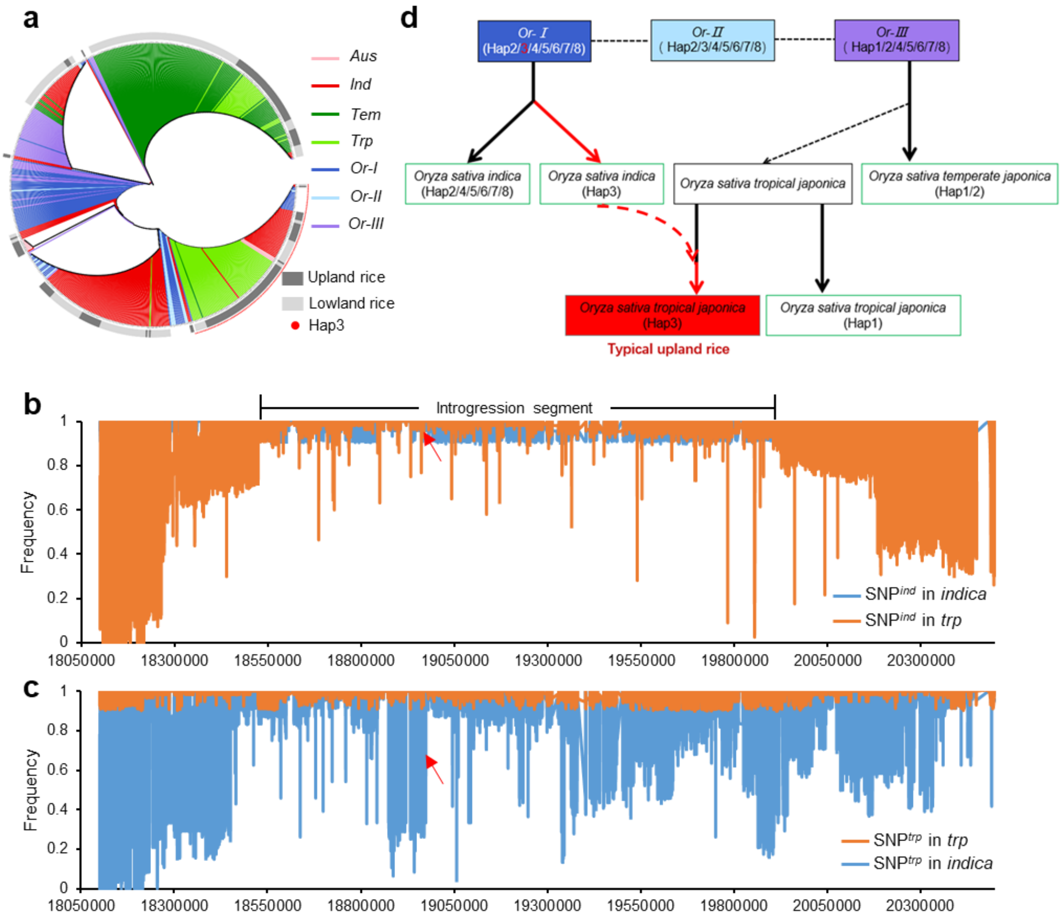

Supplementary Fig. 19. The origin and spread of *DROT1*.

a Phylogenic tree generated from 545 rice accessions consisted of 140 *O. rufipogon* and 405 *O. sativa*. The branch colors in the inner circle indicate different rice subgroups. The dark gray strip around the circle indicates upland rice, while light gray strip indicates lowland rice. Red dot in the outermost circle represents Hap3. **b, c** Introgression of Hap3 from *indica* into tropical *japonica*. Specific SNPs (SNP frequency > 0.9 in a specified subgroup) were identified in the physical region of 18.1-20.5 Mb on chromosome 10. *Indica*-specific SNPs (SNP^{ind}) were identified using 23 *indica* accessions containing $DROT1^{Hap3}$, and almost all these SNPs in tropical *japonica* share a highly identical region with *indica* from 18.5 Mb to 19.9Mb (**b**). Tropical-specific SNPs (SNP^{trp}) were identified from 57 tropical *japonica* accessions with Hap3 in *DROT1*, but the distribution of these SNPs in *indica* is at a lower frequency (**c**). **d** Schematic diagram of the hypothetic origin and spread of *DROT1*. Source data are provided as a Source Data file.

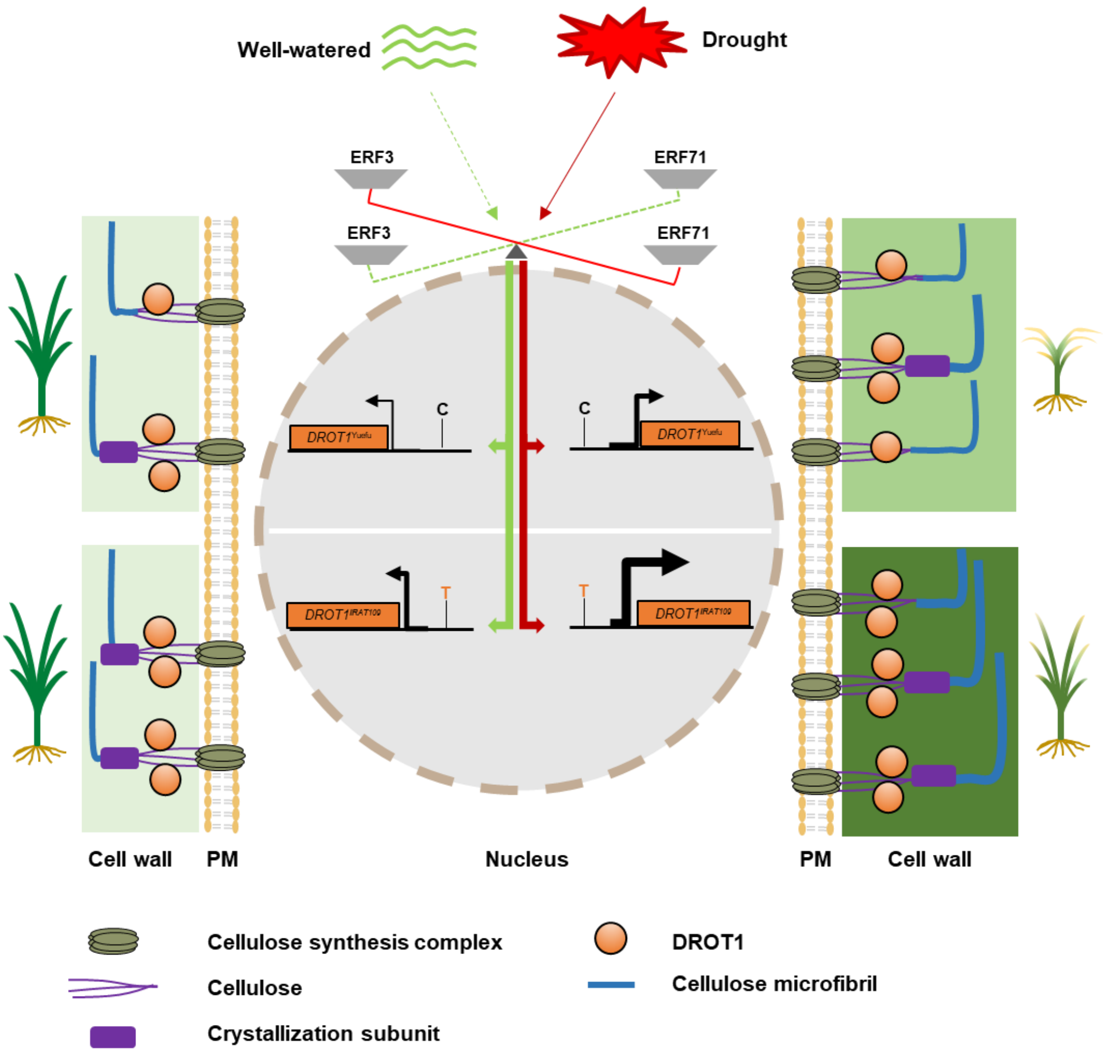

Supplementary Fig. 20. Proposed model for *DROT1* regulating drought resistance.
Under well-watered conditions, the coordinated expression of *ERF3* and *ERF71* activates the expression of *DROT1*, which is responsible for cell wall assembly during normal plant growth and development. Natural variation in the promoter leads to the differentiated expression of *DROT1*, which significantly changes the cell wall crystallinity.

Under long-term drought conditions, the expression patterns of *ERF3* and *ERF71* are changed and rebalanced, significantly promoting the expression of *DROT1*. At the same time, the number of cellulose synthesis complexes increased and more individual glucan chains are synthesized. Therefore, induced expression of *DROT1* can improve the assembly efficiency of cellulose microfibrils, which in turn increases the cellulose content and maintains the cellulose crystallinity. The higher drought resistance of upland rice is primarily attributed to the higher expression of *DROT1*, which is caused by the functional variation in its promoter.
