# Supplementary material for: Natural variation of DROT1 confers drought adaptation in upland rice
Source: Nat Commun. 2022 Jul 23;13:4265. doi: 10.1038/s41467-022-31844-w (PMC9308802; doi:10.1038/s41467-022-31844-w)
Supplement: Supplementary file 3 — Description of Additional Supplementary Files [file 41467_2022_31844_MOESM3_ESM.pdf]

Description of Additional Supplementary Files

File Name: Supplementary Data 1
Description: Information of rice accessions and phenotypic data used in GWAS.

File Name: Supplementary Data 2
Description: List of QTLs and SNPs associated with drought resistance index and leaf rolling index by GWAS.

File Name: Supplementary Data 3
Description: Phenotypic data of seedlines of introgression lines under 15% PEG treatment.

File Name: Supplementary Data 4
Description: SNPs between IL349 and Yuefu located in the predicted gene regions of the qDR10b.

File Name: Supplementary Data 5
Description: RPKM of the 25 genes in the candidate region between upland rice and lowland rice.

File Name: Supplementary Data 6
Description: Information of 743 rice accessions used for haplotype and phylogfenetic analysis.

File Name: Supplementary Data 7
Description: Primers used in this study.
