# Supplementary material for: Natural variation of DROT1 confers drought adaptation in upland rice
Source: Nat Commun. 2022 Jul 23;13:4265. doi: 10.1038/s41467-022-31844-w (PMC9308802; doi:10.1038/s41467-022-31844-w)
Supplement: Supplementary file 11 — Reporting Summary [file 41467_2022_31844_MOESM11_ESM.pdf]

nature portfolio

Corresponding author(s): Zichao Li

Last updated by author(s): Jun 23, 2022

Reporting Summary

Nature Portfolio wishes to improve the reproducibility of the work that we publish. This form provides structure for consistency and transparency in reporting. For further information on Nature Portfolio policies, see our Editorial Policies and the Editorial Policy Checklist.

Statistics

For all statistical analyses, confirm that the following items are present in the figure legend, table legend, main text, or Methods section.

n/a	Confirmed	
☐	☒	The exact sample size (*n*) for each experimental group/condition, given as a discrete number and unit of measurement
☐	☒	A statement on whether measurements were taken from distinct samples or whether the same sample was measured repeatedly
☐	☒	The statistical test(s) used AND whether they are one- or two-sided<br>*Only common tests should be described solely by name; describe more complex techniques in the Methods section.*
☒	☐	A description of all covariates tested
☐	☒	A description of any assumptions or corrections, such as tests of normality and adjustment for multiple comparisons
☐	☒	A full description of the statistical parameters including central tendency (e.g. means) or other basic estimates (e.g. regression coefficient) AND variation (e.g. standard deviation) or associated estimates of uncertainty (e.g. confidence intervals)
☐	☒	For null hypothesis testing, the test statistic (e.g. F, t, r) with confidence intervals, effect sizes, degrees of freedom and P value noted<br>*Give P values as exact values whenever suitable.*
☒	☐	For Bayesian analysis, information on the choice of priors and Markov chain Monte Carlo settings
☒	☐	For hierarchical and complex designs, identification of the appropriate level for tests and full reporting of outcomes
☒	☐	Estimates of effect sizes (e.g. Cohen's d, Pearson's r), indicating how they were calculated

Our web collection on statistics for biologists contains articles on many of the points above.

Software and code

Policy information about availability of computer code

Data collection	For collecting the images of Fluorescence in the root epidermis, ZEISS ZEN blue (Version 3.1) software was used.<br>All Fourier transform infrared (FTIR) images were taken using Spectrum IMAGE R1.7.1 Software (PerkinElmer).
Data analysis	The fast NNLS algorithm was carried out with InSituAnalyze for in situ characterizing the concentration of target component. Data from other experiments were analyzed by R 3.0.1, MEGA 6, TASSEL5.0, SPSS 20, Origin V9.1, GraphPad Prism 8 and ImageJ v1.8.0.

For manuscripts utilizing custom algorithms or software that are central to the research but not yet described in published literature, software must be made available to editors and reviewers. We strongly encourage code deposition in a community repository (e.g. GitHub). See the Nature Portfolio guidelines for submitting code & software for further information.

Data

Policy information about availability of data

All manuscripts must include a data availability statement. This statement should provide the following information, where applicable:

- Accession codes, unique identifiers, or web links for publicly available datasets
- A description of any restrictions on data availability
- For clinical datasets or third party data, please ensure that the statement adheres to our policy

Data supporting the findings of this work are provided in the paper and its Supplementary Information/Source Data files. The genetic materials supporting the findings of current study are available from the corresponding authors upon request. Source data are provided with this paper.

Field-specific reporting

Please select the one below that is the best fit for your research. If you are not sure, read the appropriate sections before making your selection.

☒ Life sciences ☐ Behavioural & social sciences ☐ Ecological, evolutionary & environmental sciences

For a reference copy of the document with all sections, see nature.com/documents/nr-reporting-summary-flat.pdf

Life sciences study design

All studies must disclose on these points even when the disclosure is negative.

Sample size	Required experimental sample size for GWAS was estimated based on our previous study on rice collections (Zhao et al., 2018) and other study on GWAS for japonica population (Yano et al., 2016). These samples include both upland and lowland rice from different geographical regions, which represents a broad genetic diversity of japonicas.<br>Ref:<br>Zhao, Y. et al. Loci and natural alleles underlying robust roots and adaptive domestication of upland ecotype rice in aerobic conditions. PLoS Genet. 14, e1007521 (2018).<br>Yano K. et al. Genome-wide association study using whole-genome sequencing rapidly identifies new genes influencing agronomic traits in rice. Nat Genet. 2016 Aug;48(8):927-34.
Data exclusions	No data were excluded from our analysis.
Replication	All experiments were successfully repeated at least three times and we have stated it in figure legends.
Randomization	For drought treatment, germinated seeds with similar vigor were randomly selected and planted in the pots. For the investigation of drought resistance in the field conditions, dry seeds were directly sown and germinated seedlings were thinned out to retain plants with similar growing performance. The plants in a single line were randomly selected for the investigation of plant height and biomass.
Blinding	Data collection and analysis were performed in a blinded manner.

Reporting for specific materials, systems and methods

We require information from authors about some types of materials, experimental systems and methods used in many studies. Here, indicate whether each material, system or method listed is relevant to your study. If you are not sure if a list item applies to your research, read the appropriate section before selecting a response.

Materials & experimental systems

n/a	Involved in the study	
☐	☒	Antibodies
☒	☐	Eukaryotic cell lines
☒	☐	Palaeontology and archaeology
☒	☐	Animals and other organisms
☒	☐	Human research participants
☒	☐	Clinical data
☒	☐	Dual use research of concern

Methods

n/a	Involved in the study	
☒	☐	ChIP-seq
☒	☐	Flow cytometry
☒	☐	MRI-based neuroimaging

Antibodies

Antibodies used	Anti-Flag antibody was used in ChIP assay. (Sigma-Aldrich, A2220, M2, lot number:SLCD7154)
Validation	Anti-flag M2 affinity gel is a mouse monoclonal antibody covalently linked to agarose. The antibody can bind FLAG at the n-terminal, Met-N terminal, C-terminal and internal sites of the fusion protein. Binding is calcium independent. The antibody is commercially available from the manufacturer. For more details, please see https://www.sigmaaldrich.cn/CN/zh/product/sigma/a2220.
